# Remote Pain Monitoring Using Fog Computing for e-Healthcare: An Efficient Architecture

**DOI:** 10.3390/s20226574

**Published:** 2020-11-18

**Authors:** Syed Rizwan Hassan, Ishtiaq Ahmad, Shafiq Ahmad, Abdullah Alfaify, Muhammad Shafiq

**Affiliations:** 1 Department of Electrical Engineering, The University of Lahore, Lahore 54000, Pakistan; ishtiaq.ahmad@ee.uol.edu.pk; 2 Industrial Engineering Department, College of Engineering, King Saud University, P.O. Box 800, Riyadh 11421, Saudi Arabia; ashafiq@ksu.edu.sa (S.A.); aalfaify@ksu.edu.sa (A.A.); 3 Department of Information and Communication Engineering, Yeungnam University, Gyeongsan 38541, Korea

**Keywords:** fog computing, cloud computing, remote pain monitoring, e-healthcare, IoT

## Abstract

The integration of medical signal processing capabilities and advanced sensors into Internet of Things (IoT) devices plays a key role in providing comfort and convenience to human lives. As the number of patients is increasing gradually, providing healthcare facilities to each patient, particularly to the patients located in remote regions, not only has become challenging but also results in several issues, such as: (i) increase in workload on paramedics, (ii) wastage of time, and (iii) accommodation of patients. Therefore, the design of smart healthcare systems has become an important area of research to overcome these above-mentioned issues. Several healthcare applications have been designed using wireless sensor networks (WSNs), cloud computing, and fog computing. Most of the e-healthcare applications are designed using the cloud computing paradigm. Cloud-based architecture introduces high latency while processing huge amounts of data, thus restricting the large-scale implementation of latency-sensitive e-healthcare applications. Fog computing architecture offers processing and storage resources near to the edge of the network, thus, designing e-healthcare applications using the fog computing paradigm is of interest to meet the low latency requirement of such applications. Patients that are minors or are in intensive care units (ICUs) are unable to self-report their pain conditions. The remote healthcare monitoring applications deploy IoT devices with bio-sensors capable of sensing surface electromyogram (sEMG) and electrocardiogram (ECG) signals to monitor the pain condition of such patients. In this article, fog computing architecture is proposed for deploying a remote pain monitoring system. The key motivation for adopting the fog paradigm in our proposed approach is to reduce latency and network consumption. To validate the effectiveness of the proposed approach in minimizing delay and network utilization, simulations were carried out in iFogSim and the results were compared with the cloud-based systems. The results of the simulations carried out in this research indicate that a reduction in both latency and network consumption can be achieved by adopting the proposed approach for implementing a remote pain monitoring system.

## 1. Introduction

There are to be about 237.1 million wearable body devices available on the market by 2020 with an estimated reach of market share related to the healthcare industry of USD 117 billion by 2020 [1]. The data flow by healthcare applications based on such a large number of bio-sensors is approximated to be 507.5 zettabytes [2]. Presently, most of the healthcare applications are designed by connecting the Internet of Things (IoT) with cloud servers. Cloud servers provide substantial on-demand resources to process, store, and analyze this large volume of health-related data. For employing healthcare applications, among the available solutions, cloud computing is currently the most feasible one [3]. Cloud computing provides all computational and storage resources at cloud servers for the processing of data generated from healthcare IoT devices. A massive and diverse amount of data is generated by healthcare applications. However, the centralized nature of cloud architecture limits the implementation of healthcare applications on a large scale because response time from the cloud rises with an increase in the volume of data to be processed. The consequences of transmitting and processing of such a huge amount of data at a remotely located cloud server result in high latency and network utilization. However, a rapid response is required in the case of healthcare applications, so processing the real-time data of patients is necessary. There are more strict quality of service (QoS) requirements when dealing with the processing of electrocardiogram (ECG) and electroencephalogram (EEG) signals [4,5]. Therefore, cloud architecture is unable to fulfil the stringent QoS requirements for medical data.

Pain is an important parameter to detect the discomfort and illness of a patient. The results of a survey conducted through different groups of patients endorse the necessity and effectiveness of remote pain monitoring [6]. Three major limitations in the self-report procedure are non-compliance of patients to manual entry, delay in treatment, and patients who are unable to express their conditions. The major reason behind the obsolescence of the self-report method is delayed diagnosis, due to which the patients have to bear the pain for a long period. These are the factors influencing recent research on automatic pain detection schemes. Important techniques used for automatic pain detection include facial expression recognition using face video [7], physiological signal fusion [8], and facial surface electromyography (sEMG). However, some efforts have been made in designing remote pain monitoring systems by integrating cloud computing with automatic pain detection tools [9,10]. Several researchers have proposed remote pain monitoring systems using cloud computing and IoT devices. The major challenge to be addressed is the fulfillment of healthcare QoS requirements during large-scale implementation.

High latency and network usage are the key factors limiting the large-scale implementation of cloud-based remote pain monitoring systems. In [11], a remote pain monitoring system is proposed in which wireless sensor nodes and web platforms are connected through a cloud server. The authors designed a wearable bio-sensing facial mask to detect intensity of pain through analyzing sEMG and ECG signals. The cloud server receives biopotential signals from sensors and after processing and displays pain-related information in a web application for real-time monitoring. However, connecting sensor nodes directly to the cloud server results in a long delay, which is not suitable for such time-sensitive healthcare applications. Therefore, we propose a fog computing architecture-based remote pain monitoring system to overcome the inadequacies of cloud computing architecture.

The key contributions of this research work are summarized as follows:

- An efficient fog-based remote pain monitoring system is proposed, consisting of a three-tier structure. Fog nodes reside in the middle tier, implementing the fog computing concept. Fog devices process the biopotential signals and transmit the pain information to the web servers via gateway devices.
- The parameters under consideration are execution cost, latency, and network consumption. The proposed architecture reduces these factors, making the proposed system most suitable for health-related applications. Moreover, it ensures real-time monitoring of patients and rapid medical assistance provisioning by minimizing the time spent from pain detection to display in the web application. The proposed architecture not only reduces time but also reduces the data to be transmitted to the cloud by discarding the unwanted data at the fog nodes.
- Simulations are performed on different scales for appraising the proposed fog-based remote pain monitoring architecture. The results of the comparison performed between cloud architecture and proposed architecture validate the superiority of the proposed architecture in terms of execution cost, delay, and network consumption.

The rest of this paper is organized as follows. Section 2 presents the background information of cloud and fog computing architectures. Recent research work related to cloud- and fog-based healthcare systems is presented in Section 3. The proposed architecture for remote pain monitoring is explained in Section 4. Section 5 describes the simulation setup and results obtained in this research. Discussion on the results and comparison with other systems is presented in Section VI. To summarize this research, a conclusion is presented in Section 6.

## 2. Background

Cloud computing has emerged as the most feasible solution for the development of IoT and big data applications by providing resourceful cloud servers for storage and processing. High latency, extra network utilization, energy efficiency, and QoS are challenges arising due to rapid growth in data traffic [12]. These problems do not allow the implementation of time-sensitive applications on cloud computing architecture. Fog computing architecture consumes less network bandwidth with has low latency to offer improved quality of experience (QoE). By offloading applications from the cloud to fog nodes, a 41% power reduction was achieved in a theoretical built-in model [13]. To satisfy the requirements of the latency and network load of sensitive and sophisticated applications, fog computing distributes computational resources near to the edge devices [14,15].

Delay in cloud-centric healthcare applications increases when deployed on a large scale [16], thus failing to attain real-time data provision for time-sensitive healthcare applications [17]. Latency requirements to maintain QoS in E-healthcare services are presented in Table 1 [18,19]. Cisco introduced the idea of fog computing in January 2014 to resolve the high network utilization and latency issues caused in cloud-based implementations [20].

Fog computing has a distributed architecture to reduce the load on the cloud. Fog computing distributes the cloud resources throughout the network by introducing fog devices with limited resources between cloud and edge devices [21,22]. All devices with limited storage and processing capability come under the definition of a fog node. The main goal of fog computing is to provide services with less latency between cloud and end devices [23].

Different researchers have proposed fog-based architectures in creating different applications that are more effective, secure, and cost-efficient. A multi-level fog-based architecture was proposed by Chen et al. [24] which enables seamless service sharing at network edges between cross-domain IoT applications. Wang et al. [25] proposed a Gini coefficient-based fog computing nodes selection algorithm (GCFSA) to get optimized computational resource allocation and off-loading decisions for maximizing the revenue of user equipment (UE) in mobility-aware three-tier fog architecture. To enhance privacy and secure communication in fog-based vehicular ad hoc networks, Ma et al. [26] designed a new authenticated key agreement protocol. By utilizing cloud and fog resources to provide network virtualization, edge computing, and other IoT services to the end user by telecommunication network operators, a fog-based architecture was proposed by Vilalta et al. [27].

Designing applications in the fog computing paradigm reduces frequent data transmissions between cloud and edge devices, assuring low latency and minimum network utilization. Research performed in [28,29,30] concludes that reduced latency is offered by fog computing models as compared to the cloud. Fog nodes are distributed throughout the network, providing scalability and mobility [31]. Consuming fog node resources to reduce the burden on the cloud significantly reduces network usage [32]. To resolve the resource allocation problem and to achieve cost efficiency, Jia et al. [33] proposed a double-matching computing resource allocation strategy in three-layer fog networks. To address the dynamic offloading and resource allocation issues in multi-layer fog computing networks, Gao et al. [34] proposed predictive offloading and resource allocation (PORA), which achieves low latency and optimal power consumption.

Fog nodes are geographically distributed throughout the network with locally available enough computing power to provide services to a variety of heterogeneous devices [35]. In this paper, we proposed to employ fog computing architecture to implement a remote pain monitoring system. An adaptable structure, low latency, and service provision near to the edge make fog computing the most suitable candidate to satisfy QoS requirements of real-time applications [36].

The proposed remote pain monitoring system makes real-time information about the pain conditions of patients available in the web portal using digital signal processing techniques and fog computing architecture. To ensure immediate medical relief to patients, the pain-related information has to be processed to make it available for remote monitoring purposes on a real-time basis. Therefore, it is desirable to achieve minimum latency and network usage. Fog computing can effectively resolve these issues by providing data processing and storage capacity near to the end devices and consequently reducing the load on the cloud [37].

In our proposed architecture, fog nodes are located to process biopotential signals of patients in hospitals to gather their pain-related information. A web application is linked with the system to present the pain-related information for remote monitoring. The platform used for the evaluation of the proposed architecture is iFogSim. Fog computing-based and cloud computing-based models for remote pain monitoring system are created and compared on different scales. The result of these simulations confirms that the proposed remote pain monitoring architecture is more effective than cloud-based architecture in terms of network load and latency.

## 3. Related Work

We briefly define the state-of-the-art healthcare monitoring systems pertinent to the cloud- and fog-based architectures in the following section.

In [38], the authors discussed different strategies for interconnecting healthcare applications and articulate the approach of implementing them in the healthcare system using mobile cloud computing. The benefits of deploying healthcare applications using cloud infrastructure are also part of this article. They designed a fall detection system for elderly people using cloud computing architecture. Tejaswini et al. [39] proposed remote pain monitoring based on cloud architecture for newborn infants to reduce the mortality rate. Infant crying is a pathological tool used as an indicator of pain in this research. Pattern classification is achieved by using support vector machine (SVM)-based neural networks. The ThingSpeak IoT platform and mobile devices are used to link clinician and nurses. In [11], the authors proposed a cloud-based remote pain monitoring architecture in which the cloud works as a bridge between IoT devices and a web application. A low-energy wearable bio-sensing mask was designed to capture sEMG and ECG signals, which were further processed to evaluate pain conditions. A web application was developed to present data for real-time remote pain monitoring. The same architecture was employed in [40] for remote monitoring of persistent vegetative state (PVS) patients through analyzing real-time signals related to facial expressions.

Mobile cloud computing is used by the authors in [41] to provide ubiquitous healthcare services in smart cities that require collecting and processing patients’ data anytime and anywhere. Network delay, high bandwidth consumption, and reliability are major hurdles in the implementation of futuristic healthcare applications. To address these issues, the authors proposed a healthcare structure, UbeHealth. To provide improved QoS, the proposed system is based on four layers and three major components. The first layer consists of healthcare professionals for remote supervision. The second layer of the architecture comprises cloudlets on which future network traffic is predicted and this information is used to maintain QoS by adapting the data rate accordingly. The network layer predicts the application that sends data using deep learning techniques to adjust the connection according to the requirements of the application to retain QoS. Finally, the cloud layer consists of resourceful cloud servers to provide data processing and storage facilities for various healthcare applications.

Rahmani et al. [16] explained the importance of locating gateway nodes near to the edge in the architecture to offer advanced-level services. To cope with the challenges involved in the implementation of ubiquitous healthcare systems, the authors proposed a fog-based architecture. To evaluate the performance of the proposed fog-based architecture, a prototype of an early warning health monitoring system was developed. The issues involved in the implementation of healthcare applications using mobile cloud architecture are discussed by Farhani et al. [42] and the fog computing paradigm was proposed to deal with these issues and to facilitate efficient network utilization in such applications. Negash et al. [43] proposed a fog-based healthcare application that consists of three tiers. The first tier of the proposed architecture consists of various sensors to detect different signals related to patients, health, the environment, and activities. The fog layer resides between the cloud and sensor layer which is responsible for the compression of received sensed data and transmitting them to the cloud for further processing. Gaigawali and Chaskar [44] structured a cloud-based healthcare system for monitoring ECG and fibrillation signals into three parts. The first part consists of biopotential sensors to acquire ECG signals. The second part is based on the cloud server to provide resources to process and store the collected ECG data. The third part is a smartphone application to provide remote access. This approach improves the healthcare systems in the provision of remote ECG monitoring in terms of accuracy.

The authors described data secrecy and the improper use of advanced information and communication technologies to be among the major reasons behind the unacceptability of cloud-based healthcare applications by patients in developing countries. In [45], a social–technical design approach was used to develop a cloud-based health center in the Nigerian healthcare system. This system provides services related to remote healthcare to rural areas, which results in cost and time reductions. A specific type of mosquito bite transmits the chikungunya virus, which causes disease. Due to problems in the availability and affordability of diagnostic tests in developing countries, fog-based remote detection and monitoring in healthcare systems are emerging as a solution to these problems. The authors in [46] designed a fog-based chikungunya virus identification and diagnosis system to enable the fast rescue to prevent an outbreak. In their design, classification is performed on user data using a decision tree for the identification of infection and the instantaneous result is transferred to users through patients’ mobiles. Furthermore, temporal network analysis is performed on the users’ data collected from the vicinity to detect the virus outbreak state.

iFogSim is a toolkit to simulate IoT applications in cloud and fog computing architectures. Several researchers have used iFogSim to evaluate their research work. In [47], the authors briefly explained the architecture and steps involved in the modeling and simulation of fog-based applications using iFogSim. Dar et al. [28] proposed an IoT-based disaster management system and compared its cloud- and fog-based implementation using the iFogSim toolkit. Qaddoura and Manaseer used [48] iFogSim to evaluate the effects of the central processing unit (CPU) speed of fog nodes on the energy consumption and end-to-end delay of the network. For optimized task scheduling, Jayasena and Thisarasinghe [49] compared the whale optimization algorithm with several heuristic and meta-heuristic algorithms in a smart healthcare application model using the iFogSim simulator tool. Fog computing-based architecture for efficient car parking is proposed in [50] and the result of simulations performed in iFogSim show that network consumption and latency in the proposed architecture is less than in the cloud-based architecture. Mahmud et al. [51] have labeled iFogSim as the most effective tool among the available simulators for simulating applications in fog computing architectures. Fang and Ma [52] proposed an application module placement and task scheduling strategy based on a heuristic dynamic task processing algorithm and the proposed schemes were evaluated in iFogSim, and the results confirm the improvement in power consumption.

## 4. Proposed Architecture

**Figure 1:**
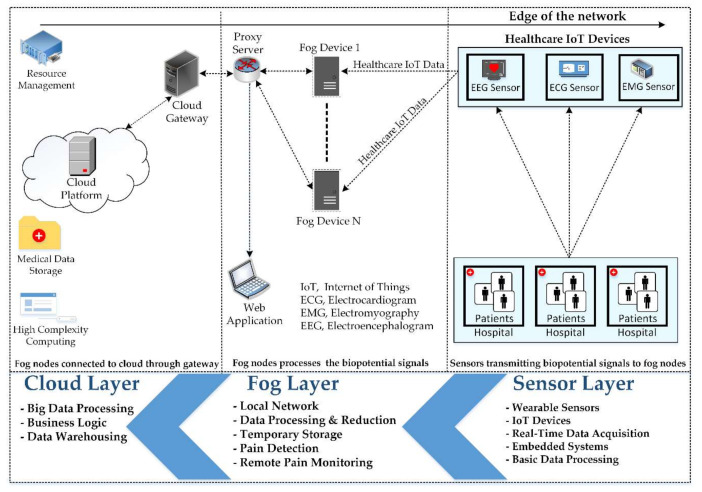
Three-tier architecture for a fog-based remote pain monitoring system.

The proposed three-layer architecture is illustrated in Figure 1. The first layer consists of biopotential sensors attached to the patients in the hospitals to detected and transmit their sEMG and ECG signals after preliminary processing to the fog nodes. The central layer of the architecture contains fog nodes to which all the sensor nodes are connected through a Wi-Fi module. The third layer is based on a cloud to which all the fog nodes are connected through a proxy server. The key purpose of the cloud server is to provide extra storage and computational resources to the system. A web application is linked with the system to provide access to pain statistics of patients for real-time monitoring, therefore minimizing the delay in providing relief to the patients and reducing the load on the paramedics. An overview and layers of the proposed architecture are defined in the following section.

### 4.1. The Sensor Layer

The first layer of the proposed architecture consists of wearable sensors that include two modules for sensing and transmitting the detected biopotential signals. To satisfy the data acquisition requirements of the EMG and ECG signals of patients, sensors are designed with passive electrodes with a battery power supply. For the transmission of data to fog nodes, the Wi-Fi module is integrated with the sensor nodes. Sensors have to continuously provide signals for pain monitoring so low power consumption is seriously considered while designing the sensor nodes to prolong battery life. The sensors deployed in our proposed system for the collection of biopotential signals have a sampling rate of 1000 samples per second, satisfying the Nyquist criteria. The sensors detect the EMG and ECG signals and transmit the sensed data to the fog nodes where they are further processed as defined in [11].

### 4.2. The Fog Layer

**Figure 2:**
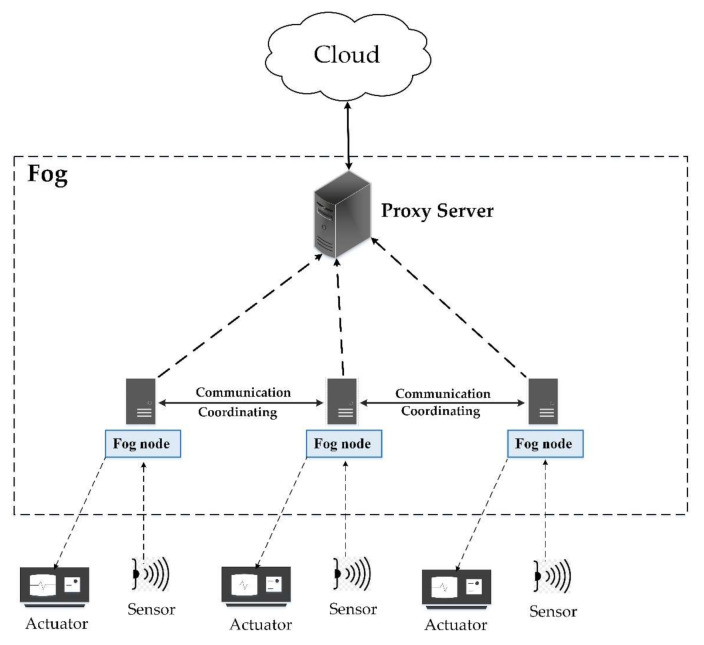
Interconnection of cloud server, fog devices, and sensors.

**Figure 3:**
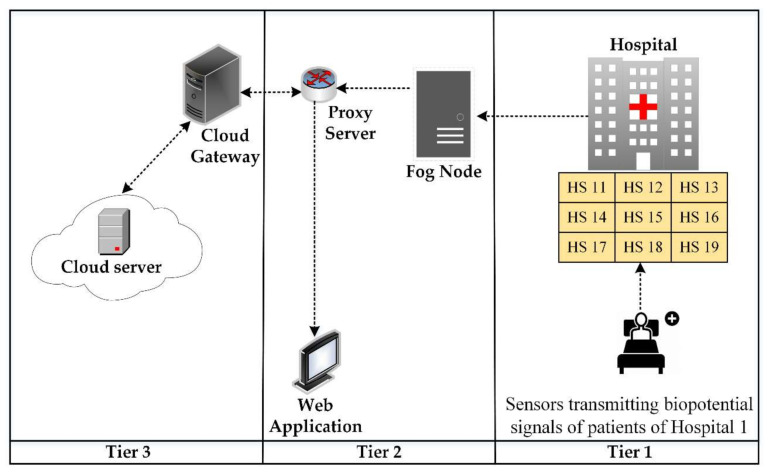
The architecture of the remote pain monitoring system for one hospital.

This layer exists between the sensor and the cloud layer. The fog layer consists of fog nodes to collect biopotential signals from sensors to perform further processing for the identification of pain. Among most of the prominent features of fog computing, interoperability is a significant one. Interoperability deals with a diverse variety of IoT devices, as shown in Figure 2. Each fog node contributes some of its local resources to interconnect with neighboring fog nodes to fulfill their processing and storage requirements [53]. In our proposed framework, we do not consider the latency factor in communication between the fog nodes, considering it an advantage of fog computing interoperability features [50]. To differentiate among different patients, specific indexes, for example, HS11 defining patient 1 of hospital 1, are used as shown in Figure 3. Fog nodes process the incoming biopotential signals using their local resources and transfer the status related to pain to the web application for remote pain monitoring. For a specific time, data are temporarily stored in the local storage, then the fog node transfers this data to the storage module located at the cloud server for maintaining the medical history of patients. Fog computing architecture provides resources to collect, process, and transmit data from the edge to the web server, enabling real-time remote pain monitoring.

### 4.3. The Cloud Layer

The third layer in our proposed architecture is the cloud layer which comprises the cloud server. The main purpose of the cloud is to provide extra storage capacity. The storage module is embedded in the cloud server, which is used for storing and updating pain-related data for record-keeping. Fog nodes are connected to the cloud via a proxy server. Fog nodes periodically submit the biopotential signal data to the cloud server after consuming these data for remote pain detection. In our proposed model, the cloud server is bypassed when computational resources available at fog nodes fulfill the processing requirements of incoming biopotential data, thus reducing the additional delay.

### 4.4. Overview

The proposed fog-based remote pain monitoring system consists of biopotential sensors, Wi-Fi modules, fog nodes, a web application, and a cloud server. The sensor node continuously detects the EMG and ECG signals of the patients in hospitals using electrodes. To detect pain using the facial action coding system [54], different facial muscles under the monitoring of the sensor nodes are frontalis, corrugator, orbicularis oculi, levator nose, zygomaticus, and risorius. Subsequently, fog nodes detect the pain by applying different digital signal processing and filtering techniques on the perceived data, as presented in [11]. Fog nodes update the pain-related information in the web application for remote monitoring. Multiple sensors are employed in hospitals to monitor all the patients. In our proposed architecture, one fog node per hospital is deployed to analyze the data received from the sensors to detect pain. Moreover, fog nodes make this pain information available for remote monitoring by transmitting it to a web application. The patient record is periodically updated in the database placed in the cloud server.

**Figure 4:**
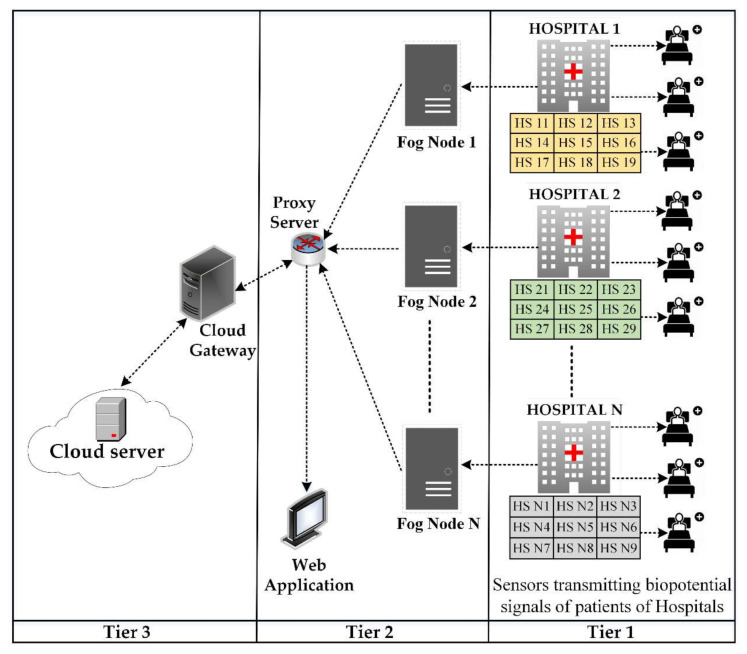
The architecture of the remote pain monitoring system for multiple hospitals.

Cloud computing has enough resources to store and process the data. However, repeated communication between sensors and the cloud produces excessive delays with high network consumption. Therefore, employing cloud computing architecture for this type of application is not desirable. Introducing a fog computing layer in between the sensor and cloud layer reduces the latency, as fog nodes provide extra capacity to process the biopotential signals close to the edge of the network. The transmission of detected pain information directly from fog nodes to the web server reduces latency and network consumption. The architecture proposed in this paper avoids the cloud server while transmitting detected pain information from fog nodes to the web application, thus offering reduced network consumption and low latency. Signal processing tasks for achieving pain information are performed at the fog nodes. Our proposed fog computing-based architectures for single and multiple hospitals are presented in Figure 3 and Figure 4, respectively.

In Figure 3, patients of only one hospital are to be monitored, hence there is a single fog node connecting sensors with the cloud and web server. On the other hand, Figure 4 presents the scenario where multiple fog nodes are deployed to monitor patients of multiple hospitals. In the first case, the fog node has to send data of a single hospital to the cloud and web server. However, in the second case, combined data of all the hospitals have to be delivered to the cloud and web server. In both cases, each fog node has to monitor a fixed number of patients. Therefore, latency and network consumption are the same on each fog node but there is an increase in time and network utilization when data from all the fog nodes have to be uploaded on the cloud and web server simultaneously.

**Figure 5:**
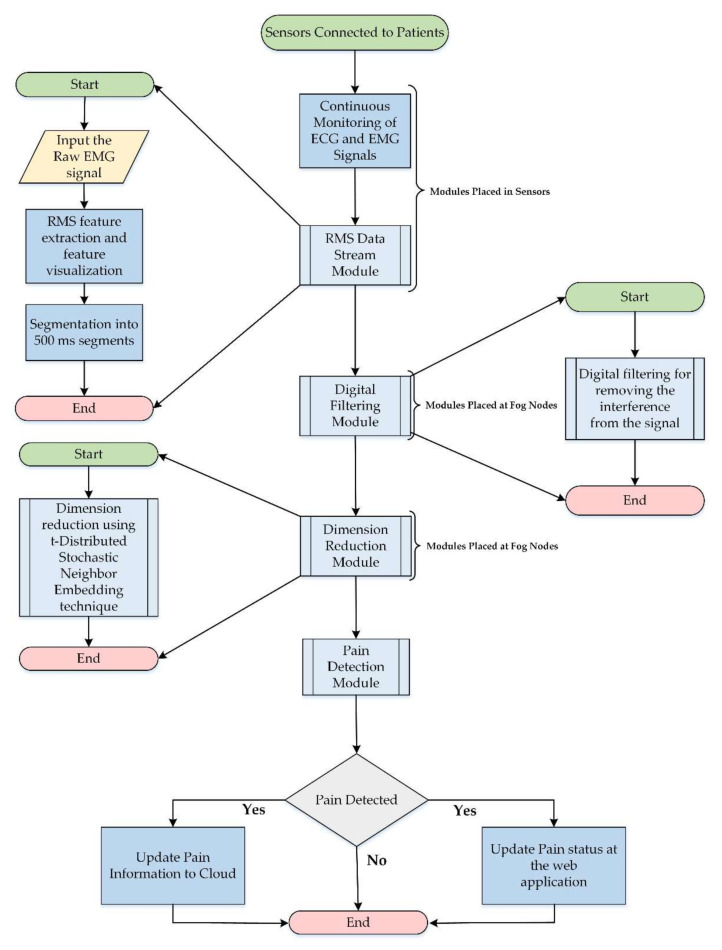
Flow diagram of proposed fog-based remote pain monitoring system.

At the start, all the sensors are initialized for the acquisition of EMG and ECG signals of patients. After the signal acquisition phase, biopotential signals are transferred to fog nodes, where the pattern recognition of facial expressions is achieved using root mean square (RMS) feature extraction and visualization techniques. Later, the signal is segmented and dimension reduction is performed [55,56]. Processed signals are transferred to the web application through a web server for remote pain monitoring. Finally, data are submitted to the cloud for record-keeping. The processes involved in the execution of a remote pain monitoring system are presented in the form of a flowchart in Figure 5.

## 5. Simulation Setup and Results

In our simulations, different scenarios are evaluated, comprising a different number of biopotential sensors to detect sEMG and ECG signals of the patients. These captured biopotential signals are frequently transmitted to the fog nodes. Further processing is performed by the fog nodes to detect pain status and they transmit the pain information to the web application and cloud for remote monitoring and record updates, respectively. The connection of fog node(s) with the web application and the cloud server is established via a proxy server. For simulating and evaluating our scenarios in terms of latency, execution cost, and network consumption, we used the iFogSim toolkit.

Variables of hospitals and sensors are created in the simulations. In our scenarios, there are four hospitals and one fog node is assigned to each hospital. Initially, four sensors are attached to each fog node to capture the biopotential signals of the patients. Fog nodes are connected with the cloud and web application through a proxy server. We created sensors in the simulation environment according to the policies of [1]. Scenarios are simulated with an increasing number of sensor nodes per fog device to calculate the latency and network consumption. Table 2 illustrates the biopotential sensor configuration used in our simulations.

**Figure 6:**
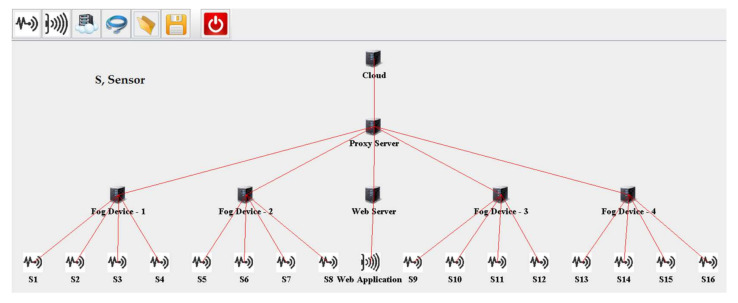
iFogSim topology of the proposed fog-based remote pain monitoring system.

The topology created in iFogSim to evaluate fog computing-based architecture is defined in Algorithm 1 and depicted in Figure 6. Four fog nodes are created and each fog node is initially linked with four sensor nodes. The metrics under observation are latency and network utilization. We embedded an RMS data stream module in the sensors to capture the biopotential signals of the patients. To process the biopotential signals for pain detection, the digital filtering module and dimension reduction module are embedded in fog nodes. Moreover, the pain detection module runs on the web server to present pain information in the web application. To define data dependency among the modules of the proposed remote pain monitoring system, edges are created between the application modules, as shown in Algorithm 1. In iFogSim, the tasks are described in the form of tuples. Tuples are generated by sensors and processed at the application modules (virtual machines) placed at the fog nodes. Each virtual machine (VM) executes a specific type of tuple. Fog devices provide resources to these application modules for performing their computations. A first come first served (FCFS) scheduling scheme is used in our simulations that assigns resources to modules in the order of entry.
**Algorithm 1** Fog-based remote pain monitoring system with first come first served (FCFS) scheduling.
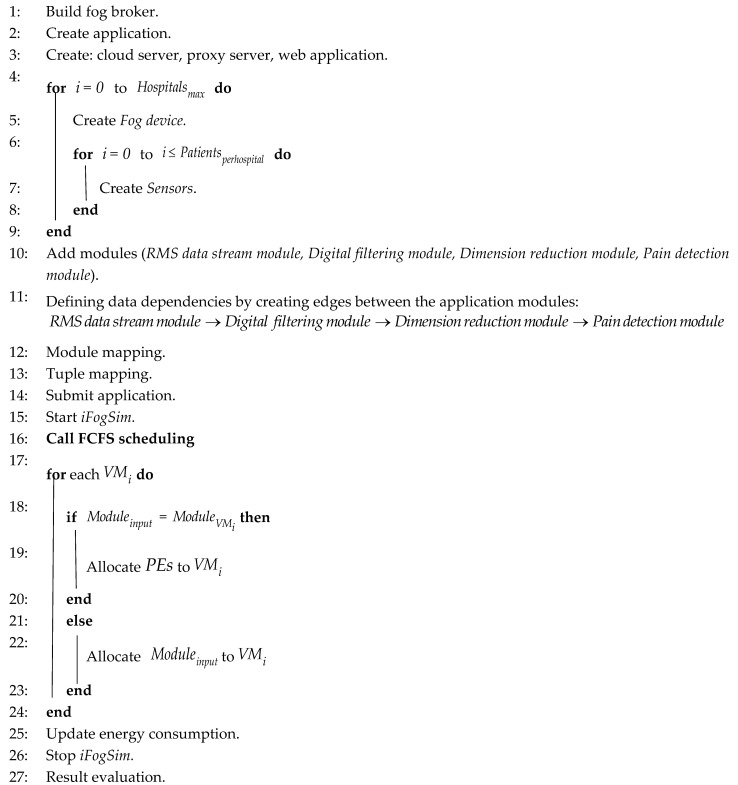


To evaluate the performance on a large scale, the number of sensors per fog device is increased in each scenario and the task of pain detection is performed for a growing number of patients using the resources of each fog node. An increase in the number of sensors attached to a fog node eventually increases the processing load on that particular fog node, causing a rise in the latency and network consumption of that specific fog node. The benefit of fog-based processing is a decrease in the computational burden on the cloud. On the contrary, connecting the sensors and web server directly to the cloud results in a longer delay and excessive network utilization.

**Figure 7:**
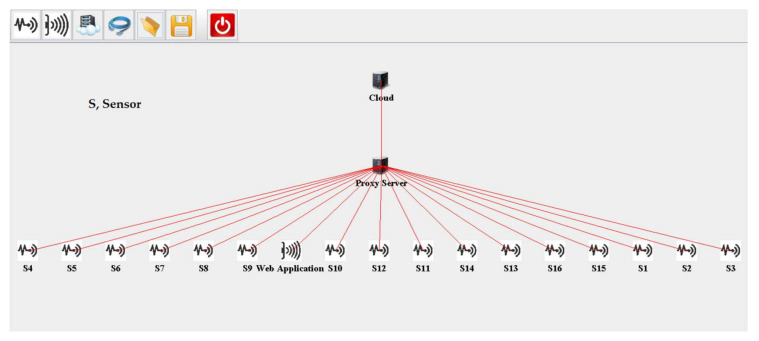
iFogSim topology of the cloud-based remote pain monitoring system.

Table 3 illustrates the values of parameters used in the creation of a cloud server, proxy server, web server, and fog nodes while simulating our scenarios. The parameters include the central processing unit (CPU) computing capacity in million instruction per second (MIPS), level in the architecture, bandwidth of uplink, random access memory (RAM), downlink bandwidth, the rate per million instructions processing, busy power, and idle power. For evaluating cloud-based implementation in iFogSim, the web application and sensors are attached to the cloud via a proxy server, as shown in Figure 7. Biopotential signals detected by the sensors are transferred to the cloud, where they are processed and information related to pain is transferred via remote access to the web application through a web server linked to the cloud. For evaluating latency and network consumption, the sensors attached to the cloud are gradually increased.

### 5.1. Execution Cost

In our scenarios, there are M smart hospitals (SHs) ($M=\{m_{1},m_{2},m_{3},...,m_{M}\}$) and the total processing load of the system to process the biopotential signals of the patients in a given t is the sum of the processing load of all the hospitals ($L_{M}^{t}=\left\{l_{m1}^{t}+l_{m2}^{t}+l_{m3}^{t},...,l_{mM}^{t}\right\}$). The total delay ($D_{s}^{t}$) of the system caused due to the processing of biopotential signals can be calculated as

(1) $$D_{s}^{t}=B_{p}^{t}\cdot \sum_{m=1}^{m=M}L_{m}^{t}$$

where $B_{p}^{t}$ represents time consumed in capturing and processing of biopotential signals of a patient.

Equation (2) is used to calculate the total time consumed ($T_{f}^{t}$) in sensing the biopotential signals according to the availability of pain status in the web application in fog-based architecture [50].

(2) $$T_{f}^{t}=D_{s}^{t}+D_{f}^{t}+D_{ftw}^{t}$$

where $D_{f}^{t}$ is the time consumed in transferring of sensed data from sensors to fog nodes and $D_{ftw}^{t}$ is the time consumed to transfer pain information from fog node to web platform. Equation (3) is used to calculate the total delay ($T_{c}^{t}$) offered by the cloud-based architecture. In cloud-based remote pain monitoring architecture, $D_{c}^{t}$ is the time consumed by transferring the sensed data from sensors to the cloud server and $D_{ctw}^{t}$ is the time consumed by displaying pain information on the web platform. Total delay ($T_{c}^{t}$) offered by the cloud-based architecture can be calculated as

(3) $$T_{c}^{t}=D_{s}^{t}+D_{c}^{t}+D_{ctw}^{t}$$

At any time t, network consumption for the cloud and proposed fog-based architecture is calculated using Equations (4) and (5),

(4) $$N_{c}^{t}=\delta \left(D_{s}^{t}+D_{c}^{t}+D_{ctw}^{t}\right)$$

(5) $$N_{f}^{t}=\delta \left(D_{s}^{t}+D_{f}^{t}+D_{ftw}^{t}\right)$$

where δ is the length of data encapsulated in the tuple.

Fog devices receive and process the data sensed by the edge devices by using the locally available resources. The data which require more computational and storage resources than those available at the fog nodes are transferred to the cloud. This fog-based orchestration minimizes the load on the cloud, thus reducing the cost of execution. The execution of application modules at the cloud increases the total execution cost. Equations (6) and (7) are derived from [57] to calculate total execution cost (E) and reduction in execution cost ($\Delta_{E}$), respectively.

(6) $$Ε=T_{c}+\left(C_{i}\cdot L_{time}\cdot R_{MIPS}\cdot L_{u}\cdot T_{MIPS}\right)$$

(7) $$\Delta_{E}=E_{cloud}-E_{fog}$$

where $T_{c}$ is the execution cost, $C_{i}$ is the CloudSim clock, $L_{time}$ is the last utilization update time, $R_{MIPS}$ is the rate per MIPS, $L_{u}$ is the last utilization, and $T_{MIPS}^{}$ is the total MIPS of the host.

**Figure 8:**
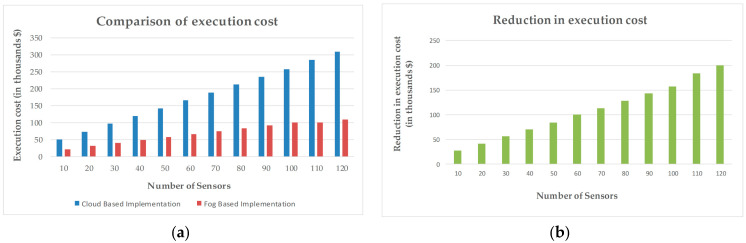
Cost of execution in the cloud. (**a**) Comparison of execution cost between cloud- and fog-based implementation. (**b**) Reduction in execution cost using proposed approach.

Fog architecture provides processing resources near to the edge of the network. Fog devices collect and process the biopotential data coming from the sensors. The data demanding higher storage and processing resources than available at the fog node are transferred to the cloud. The execution cost at cloud includes the cost required for the execution of application modules in the cloud [57,58,59]. Fog computing adaptation reduces the execution cost in the cloud by minimizing the amount of data transfer to the cloud server. The tasks are described in the form of tuples in iFogSim. Tuples are generated by sensors and processed at the application modules placed at the fog nodes. An increase in the number of sensors increases the tuples to be processed at the modules. Figure 8a illustrates the comparison of the cost of execution at the cloud for both fog- and cloud-based remote pain monitoring systems. The cost of execution in the cloud for the proposed approach is lower than the cloud-based system, indicating the reduction of data to be executed at the cloud. The reason for this data reduction at the cloud is the engagement of fog nodes in the system, providing an extra layer of data processing between the source of data and the cloud server. Figure 8b presents the reduction in execution cost during fog-based implementation.

### 5.2. Latency

Latency is an obligatory aspect to be reduced when implementing in real-time environments that demand high efficiency. The key advantage of fog architecture is that it tends to repetitively minimize access to the cloud by executing the required task at the fog nodes by using locally available resources to provide rapid response to the edge nodes, reducing latency.

**Figure 9:**
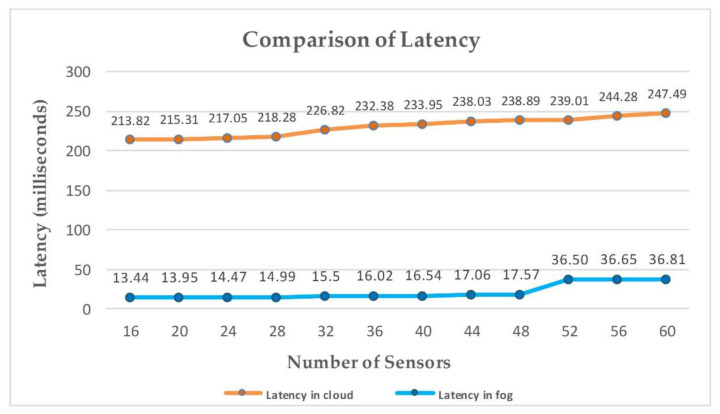
Latency comparison of proposed fog-based architecture with cloud computing architecture for implementing remote pain monitoring.

As one fog node is allocated to each hospital, sufficient processing capacity is available to process the signals of patients for detecting pain and make it available for remote monitoring in the web application within less time. We simulated all the scenarios in iFogSim to compute the results. Figure 9 compares the latency for all the scenarios for both cloud and fog architecture-based simulations. Latency in cloud-based architecture significantly increases with an increase in the number of sensors. In fog-based scenarios, the fog nodes only process the sensed data of the sensors attached to them. Contrarily, the cloud server has to process the signals sent by all the sensors so, subsequently, the latency in the cloud increases with an increase in the number of sensors.

To evaluate the fog-based system, there are four fog devices to which sensors are attached. Initially, in the first scenario, there are four sensors attached to each fog node to monitor patients. In each succeeding scenario, there is an increase of one sensor per fog device. With an increase in the number of sensors, the data to be processed at the fog devices also increase. Modules at the fog nodes have to process the data of an increasing number of sensors using locally available limited resources. Therefore, an increase in the data to be processed by the modules results in increased execution delays offered by modules. An increase in processing load on each fog device at the same instant results in an abrupt rise in the latency of the system, as depicted in Figure 9, when the number of sensors increases from 48 to 52. This rapid increase in latency of the system is the combined effect of delays offered by all fog devices. This abrupt increase in latency can be avoided by increasing the number of fog nodes in the system.

### 5.3. Network Consumption

Only cloud resources are available to process the inquiries in the case of cloud-based implementation. An increase in the number of patients to be monitored results in increased traffic towards the cloud server, thus causing increased network usage. In the case of geographically dispersed servers, a single fog node is assigned to a hospital to monitor patients of only that specific hospital. As a result, the use of the network in this situation decreases, thus providing improved throughput for the remaining traffic.

The outcomes of the simulations verified that for implementing a remote pain monitoring system, our proposed architecture, which is depicted in Figure 6, is more effective than cloud-based architecture. In the cloud-based scenario, all the sensors are attached to the cloud via a proxy server. To evaluate fog-based architecture, there are four fog devices to which sensors are attached. Initially, in the first scenario, there are four sensors attached to each fog node. In each succeeding scenario, there is an increase in the number of sensors attached per fog device. For example, the third scenario has twenty-four sensors, which means the attached number of sensors per fog device is six.

**Figure 10:**
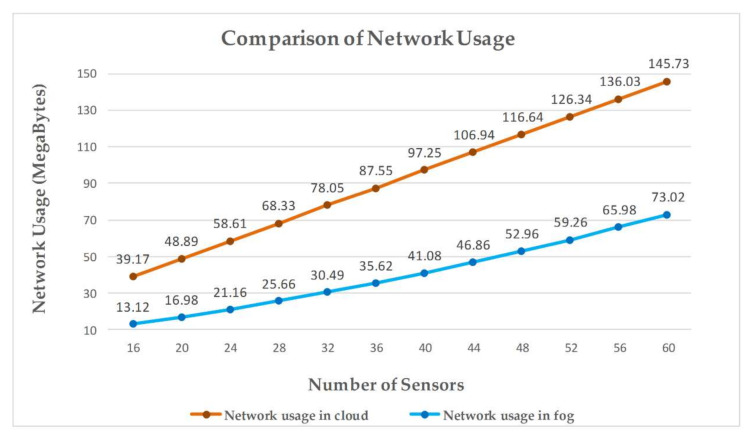
Network usage comparison of proposed fog-based architecture with cloud computing architecture for implementing remote pain monitoring.

Figure 10 presents the comparison of network usage in cloud- and fog-based scenarios. It is observed that with an increment in the number of sensors, the network utilization also increases. The reason behind the additional increase in network utilization in the case of the cloud-based environment is that all the sensors are attached to the same cloud server, which has to process all the signals coming from the sensors at the same time. On the other hand, in the fog-based implementation, an equal number of sensors are attached to each fog node to process patient data for a specific hospital. In this case, each fog node only has to process the signals of the sensors directly linked to that fog node. The results of the simulations carried out implementing both fog architecture and cloud architecture for the targeted metrics, namely latency and network usage, indicate that the proposed fog-based architecture is more effective in employing a remote pain monitoring system.

When using the fog-based architecture for remote pain monitoring, information about the pain of patients of a certain hospital can be extracted promptly and will also reduce the delay in the provision of medical assistance to the patients. The findings also enable us to understand the importance of fog computing architecture in IoT applications, where a rapid response is extremely desired. In conclusion, low latency and efficient network utilization make fog-based architecture more viable for real-time health applications.

## 6. Results and Discussion

We have presented improvement in the results by adopting a fog computing approach in different applications. To the best of our knowledge, no application using fog computing architecture for remote pain monitoring has been proposed before. To validate the effectiveness of the proposed architecture, the results are compared with the cloud-based systems that use sEMG or ECG signals for pain detection and healthcare service [11,40,60]. Execution costs in cloud, latency, and network usage are the parameters that are observed during the comparison. The results of the simulations performed on different scales validate the effectiveness of the proposed architecture for the implementation of remote pain monitoring applications as compared to the cloud.

Various simulations are performed to compare the proposed fog-based approach with the cloud-based implementation of remote pain monitoring applications. Network consumption calculated for each scenario is shown in Figure 10, which shows that the cloud-based system consumes more of the network as compared to fog architecture. In cloud-based implementation, a cloud server is used for all storage and processing tasks and, therefore, all the data from the sensors are transmitted to the cloud, resulting in high utilization of network resources. Figure 9 compares the latency for all the scenarios for both cloud and fog architecture-based simulations. Latency in cloud-based architecture significantly increases with an increase in the number of sensors, as fog nodes only process the sensed data of the sensors attached to them. Contrarily, the cloud server has to process the signals sent by all the sensors so, subsequently, the latency in the cloud increases with an increase in the number of sensors. The key advantage of fog architecture is that it tends to repetitively minimize access to the cloud by executing the required task at the fog nodes by using locally available resources to provide a rapid response to the edge nodes, reducing latency. Fog computing adaptation reduces the execution cost in the cloud by minimizing the amount of data transfer to the cloud server, as shown in Figure 8a. The reason for this data reduction at the cloud is the engagement of fog nodes in the system, providing an extra layer of data processing between the source of data and the cloud server. Figure 8b presents the reduction in execution cost during fog-based implementation.

In [11], a remote pain monitoring application was designed that detects sEMG signals of patients using sensors. Subsequently, the sensed data are transmitted to the cloud for processing and storage. Finally, the pain-related statistics are transferred to a mobile web application for remote access. Similarly, in [40], a cloud-based system for the remote monitoring of persistent vegetative state (PVS) patients using sEMG sensors was designed. In [60], a cloud-based health monitoring system was designed to monitor body temperature, oxygen saturation, and heart rate of patients. All these systems engage cloud servers for the processing and storage of biopotential data coming from patients. Moreover, web and mobile applications are linked with the cloud to offer remote monitoring services.

Cloud computing architecture provides resources in a centralized manner. Latency is a major concern in the deployment of remote health monitoring applications. Fog computing introduces a new layer comprising fog nodes near to the edge of the system. Resources available at fog nodes are limited but are sufficient for the pre-processing of the biopotential data coming from the edge of the network. Therefore, to meet the QoS requirements of e-healthcare applications, especially in terms of real-time response, the results of simulations performed in this research confirm fog computing-based deployment to be a more suitable option than the cloud. In Table 4, we briefly compare our proposed fog-based remote pain monitoring system with the existing healthcare systems. Simulation results presented in the previous section shows that a significant reduction in network consumption and execution cost can be achieved by using fog architecture as compared to the cloud.

## 7. Conclusions

To ensure the provision of medical facilities to each patient, the healthcare industry is moving towards remote health monitoring applications. Several cloud computing-based remote healthcare applications are available on the market. The key factor limiting the large-scale implementation of such applications is latency. Fog computing architecture provides an additional layer of resources near to the edge of the network. For this purpose, we proposed a fog computing-based remote pain monitoring system that collects and processes sEMG signals of patients to detect pain. In the proposed model, pain-related information is available for remote access through a web application within a minimum time, thus enabling timely medical facilitation to the patients. The result of the simulations carried out on different scales reveals that the proposed fog-based approach not only reduces latency but also minimizes the network consumption and execution cost as compared to the cloud.

The proposed approach limits the use of a single fog device for a hospital. An increase in the number of patients requires more processing resources than assigned to a fog node. Therefore, load balancing will be required to maintain the efficiency of the system. Hence, our future work includes the investigation of load balancing issues in fog computing and presenting an effective solution to resolve them. Moreover, our proposed system is just limited to pain monitoring and, in future, we are enthusiastic to design and implement a real-time fog computing-based remote healthcare system capable of monitoring multiple biostatistics related to the overall health of a patient.

## Figures and Tables

**Table 1:** Quality of service (QoS) requirements for real-time e-healthcare services.

Real-Time E-Healthcare Services	Healthcare Applications	Type of Media	Maximum Delay
Audio communication	Audio conversation between patients and doctors	Audio	<150 milliseconds one-way
Video communication	Video conferencing between patients and doctors	Video	<250 milliseconds one-way
Robotic services	Tele-ultrasonography	Control signals related to robotics	<300 milliseconds round-trip time
Monitoring services	Remote pain monitoring	Biosignal of patients gathered by sensors	<300 milliseconds for real-time ECG

**Table 2:** Biopotential sensor configuration in iFogSim simulator.

CPU Length	Network Length (bytes)	Sensor Detecting Interval
1200 million instructions	22,000 bytes	25 milliseconds

**Table 3:** Value of parameters used for cloud- and fog-based implementations.

Parameter	Cloud	Proxy Server	Web Server	Fog Node	Sensor Node
Level	0	1	2	2	3
Rate per MIPS	0.01	0.0	0.0	0.0	0.0
RAM (MB)	40,000	4000	4000	4000	1000
Idle power	16 × 83.25	83.43	83.43	83.43	82.44
Downlink bandwidth (MB)	10,000	10,000	10,000	10,000	-
CPU length (MIPS)	44,800	2800	2800	2800	500
Uplink bandwidth (MB)	100	10,000	10,000	10,000	10,000
Busy power (Watt)	16 × 103	107.339	107.339	107.339	87.53

**Table 4:** Comparison of the proposed remote pain monitoring system with the existing systems.

Reference	Paradigm	Remote Monitoring	Response Time	Cost of Execution in Cloud	Network Consumption
[61]	Cloud	Pain	Moderate	High	High
[39]	Cloud	Pain	Moderate	High	High
[62]	Cloud	Health	Moderate	High	High
[40]	Cloud	Patient	Moderate	High	High
[11]	Cloud	Pain	Moderate	High	High
Proposed System	Fog	Pain	Minimum	Low	Low
